# Impact of disease-modifying therapy on [^99m^Tc]Tc-DPD SPECT/CT markers in transthyretin cardiac amyloidosis enabled by artificial intelligence

**DOI:** 10.1007/s00259-025-07673-6

**Published:** 2025-11-29

**Authors:** Clemens P. Spielvogel, Markus Köfler, Zewen Jiang, Jing Ning, Josef Yu, David Haberl, Christina Kronberger, Michael Poledniczek, Lena Marie Schmid, David Kersting, Nikita Ermolaev, Roza B. Eslam, Michaela Auer-Grumbach, Christina Binder, Franz Duca, Christian Nitsche, Johannes Kastner, Jutta Bergler-Klein, Andreas A. Kammerlander, Christian Hengstenberg, Marcus Hacker, Raffaella Calabretta, René Rettl

**Affiliations:** 1 Division of Nuclear Medicine, Department of Biomedical Imaging and Image-guided Therapy, Medical University of Vienna, Spitalgasse 23, Vienna, A-1090 Austria; 2 Comprehensive Center for Artificial Intelligence in Medicine, Medical University of Vienna, Vienna, Austria; 3 Division of Cardiology, Department of Internal Medicine II, Medical University of Vienna, Vienna, Austria; 4 Department of Nuclear Medicine, University Hospital Essen, University of Duisburg- Essen, Essen, Germany; 5 Department of Orthopedics and Trauma Surgery, Medical University of Vienna, Vienna, Austria

**Keywords:** Transthyretin, Cardiac amyloidosis, Treatment response, SPECT/CT, Artificial intelligence

## Abstract

**Purpose:**

Transthyretin cardiac amyloidosis (ATTR-CM) is a progressive, underdiagnosed disease with high morbidity and mortality. While disease-modifying therapies (DMTs) slow progression, early treatment response markers remain scarce. This study assessed AI-quantified thoracic [^99m^Tc]Tc-DPD SPECT/CT markers as potential non-invasive biomarkers for monitoring therapeutic efficacy.

**Methods:**

This longitudinal study included ATTR-CM patients receiving DMTs (transthyretin stabilizers, RNA interference, or antisense oligonucleotides). [^99m^Tc]Tc-DPD SPECT/CT scans were acquired at baseline and after treatment (median interval 9 months, IQR 7–10). AI-driven segmentation and quantification extracted 26 markers (SUV metrics, retention index, amyloid-affected volume, and amyloid activity). Functional, clinical, and blood parameters, as well as clinical outcomes, were evaluated for their association with changes in imaging markers.

**Results:**

In 45 patients (37 ATTRwt-CM, 8 ATTRv-CM), 65% (17/26) of AI-extracted SPECT/CT markers significantly decreased after treatment (all *p* < 0.001), including SUV_max_ reductions in the left ventricle (18.6 to 14.1) and myocardium (19.5 to 15.5). None of the markers significantly increased (*p* > 0.05). Six of the imaging markers, most notably SUV_peak_ (*p* = 0.007) of the myocardium and amyloid activity of the left ventricle (*p* = 0.009), were associated with reductions in NT-proBNP. Lower values for three markers, including amyloid activity of the myocardium, retention index, and SUV_mean_ of the left atrium (all *p* = 0.016), were associated with improved NYHA class. An increase in amyloid-affected volume of the right ventricle (HR 3.19, 95% CI [1.29; 7.86], *p* = 0.005) and a decrease in right ventricular SUVmean (adjHR 0.15 95% CI [0.02;1.10], logrank *p* = 0.030) were associated with death or heart failure-associated hospitalization before and after multivariate adjustment. AI-driven analysis extracted imaging markers substantially faster and eliminated inter-rater variability.

**Conclusion:**

AI-driven [^99m^Tc]Tc-DPD SPECT/CT analysis effectively detects treatment-induced reductions in cardiac amyloid burden, offering a non-invasive biomarker for early response assessment in ATTR-CM. AI-enabled imaging markers enhance reproducibility and efficiency, providing valuable support for personalized treatment strategies as new therapeutic options for ATTR-CM become available.

## Introduction

Transthyretin cardiac amyloidosis (ATTR-CM) is a progressive and underdiagnosed condition associated with significant morbidity and mortality. It results from the deposition of misfolded transthyretin (TTR) proteins in the myocardium, leading to increased myocardial stiffness, heart failure, and ultimately death. Over recent years, disease-modifying therapies (DMTs) targeting TTR, including TTR stabilizers, ribonucleic acid (RNA) interference, and antisense oligonucleotides, have promised to slow disease progression [1–5]. However, robust and reliable markers for early treatment response remain limited, making clinical monitoring of therapeutic efficacy challenging [6–8]. With the anticipated expansion of available therapeutic options beyond TTR stabilizers, the need for reliable and non-invasive methods to assess treatment response becomes increasingly critical. As new disease-modifying therapies progress through clinical trials and regulatory approvals, identifying imaging biomarkers capable of distinguishing early therapeutic benefits will be essential for optimizing patient management. Standardized response assessments could facilitate timely treatment adjustments, improve prognostication, and ultimately enhance patient outcomes in ATTR-CM. Imaging techniques, particularly thoracic [^99m^Tc]Tc-3,3-diphosphono-1,2-propanodicarboxylic acid (DPD) SPECT/CT, have emerged as valuable tools for the diagnosis of ATTR-CM [9–11]. The ability to quantify myocardial amyloid burden through this modality has also raised interest in its potential application for treatment monitoring [12, 13]. The application of artificial intelligence (AI), particularly via segmentation-based methods presents a promising approach for extracting quantitative imaging biomarkers with high precision, while simultaneously minimizing inter-rater variability and substantially reducing the time required for the otherwise labor-intensive delineation process [11, 14, 15].

This study evaluates the impact of DMTs on [^99m^Tc]Tc-DPD SPECT/CT-derived markers quantified through AI. By analyzing changes in myocardial uptake parameters, we aim to determine their potential role as non-invasive biomarkers for monitoring therapeutic efficacy. Our findings provide insights into the ability of AI-enhanced imaging techniques to track amyloid burden over time and support personalized treatment strategies for patients with ATTR-CM.

## Materials and methods

### Study design and participants

This study was conducted within the framework of a prospective heart failure registry at a European tertiary university-affiliated hospital, which includes a dedicated amyloidosis outpatient clinic. Ethical approval was obtained from the local institutional review board (1079/2023) following the Declaration of Helsinki. Written informed consent was obtained from all participants prior to their inclusion in the registry for baseline and follow-up assessments.

Patients diagnosed with ATTR-CM between February 2019 and April 2021 were consecutively screened for study eligibility. Participants with confirmed ATTR-CM underwent baseline and follow-up assessments at the dedicated amyloidosis outpatient clinic as part of a prospective imaging study. Exclusion criteria included the inability to undergo whole-body [^99m^Tc]Tc-DPD scintigraphy (spine injury or general frailty), missing thoracic SPECT/CT imaging data at baseline or follow-up and missing treatment with ATTR-targeting therapy before the baseline assessment.

This longitudinal study included patients with confirmed ATTR-CM undergoing DMT (either tafamidis, inotersen or patisiran). Participants underwent [^99m^Tc]Tc-DPD SPECT/CT imaging at baseline, subsequently received TTR-targeted treatment within two weeks, and follow-up [^99m^Tc]Tc-DPD SPECT/CT imaging after therapy initiation. AI-based segmentation of cardiac structures was performed using a foundational deep learning segmentation model [16], enabling automated extraction of 26 quantitative markers, including standardized uptake value (SUV) metrics, retention index, amyloid-affected volume, and amyloid activity across cardiac substructures. Cardiac substructures included the myocardium and the volumes around the ventricles and atria. Imaging metrics were compared pre- and post-treatment and changes were associated with functional parameters and clinical outcomes.

### Diagnosis of transthyretin cardiac amyloidosis

[^99m^Tc]Tc-DPD scintigraphy was performed in patients with clinical evidence of cardiac amyloidosis according to the non-invasive diagnostic algorithm proposed by Gillmore and colleagues [17]. ATTR-CM was diagnosed when patients had significant cardiac tracer uptake (Perugini grade ≥ 2) alongside the absence of paraprotein or monoclonal protein detected by serum and urine immunofixation and serum free light chain assay [18, 19]. All patients diagnosed with ATTR-CM were offered the option of TTR gene sequencing, which was accepted by all individuals.

### Acquisition of imaging and clinical data

Planar whole-body images were acquired at 2.5 h and thoracic SPECT/CT at 3.0 h post intravenous administration of [^99m^Tc]Tc-DPD (mean activity: 725 ± 26 MBq) using a Symbia Intevo SPECT/CT system (Siemens Medical Solutions, Erlangen, Germany) with a low-energy, high-resolution collimator. SPECT images were acquired in a 180° configuration (64 views, 20 s per view, 256 × 256 matrix, 15% energy window around the Tc-99m photopeak at 141 keV), followed by a low-dose CT scan for attenuation correction (130 kV, 35 mAs). Image reconstruction was performed using xQUANT (eight iterations, four subsets, 3.0 mm smoothing, 20 mm Gaussian filter), standardized with a 3% NIST-traceable precision source to ensure cross-site uptake value consistency. Image acquisition parameters remained constant for baseline and follow-up examinations. Clinical and blood parameters were collected from the electronic health records of the hospital information system. Outcomes were collected from Statistics Austria and covered deaths and hospitalizations in all Austrian hospitals.

### SPECT/CT marker quantification

Overall, 26 imaging markers were quantified from each SPECT/CT image. Four types of markers were extracted, including SUV metric-based parameters, retention index, amyloid activity, and affected volume. All markers were based on initial automated delineations performed using a foundational deep learning segmentation model trained on more than 1200 expert-annotated CT images by application of the model to the CT attenuation maps of the corresponding SPECT/CT images [16]. Depending on the individual marker, delineated anatomical structures included the myocardium, left ventricle (LV), right ventricle (RV), left atrium (LA), or right atrium (RA). For SUV metric-based parameters, each anatomical structure’s segmentation mask was expanded by 10 mm to correct for potential spill-out effects, coregistration, and segmentation artifacts. Segmentation masks were then transferred from CT to the coregistered SPECT for subsequent SPECT-based quantification. SUV_max_​, SUV_mean_, and SUV_peak_ were extracted for each corresponding anatomical structure as well as for the combined atria and combined ventricles. SUV_peak_ was defined as the mean uptake within a 1 ml volume of interest (VOI) that was automatically placed to maximize the contained uptake. For the assessment of the retention index [13], the right paraspinal muscle and the vertebrae (T7-T11) were segmented using the aforementioned CT-based segmentation model with subsequent transfer to SPECT. The retention index was calculated as the ratio of the SUV_peak_ of the myocardium to the SUV_peak_ of the T9 vertebra and subsequent multiplication by the SUV_peak_ of the paraspinal muscle between T7 and T11 [13]. Amyloid activity was defined as the SUV_mean_ of the VOI multiplied by its volume. The volume was defined based on the 10 mm expanded region around the target structure to account for spill-out effects and potential inaccuracies in the registration. To determine the affected volume for each anatomical region, the SPECT image was normalized using target-to-background normalization using the inferior vena cava, which was fully automatically segmented using the aforementioned model. The coregistered CT-based segmentations for each anatomical region were dilated by 10 mm, transferred to the TBR-normalized SPECT, and subsequently thresholded for values below 1 to yield the affected volume. The code for extracting the AI-based SPECT/CT markers is installable via pip (https://pypi.org/project/spectquant).

### Reader variability assessment

To compare AI-based assessment with the time necessary for manual marker quantification and estimate inter-rater variability, three nuclear medicine physicians delineated and quantified SPECT/CT markers on a randomly sampled subset of 20 patients. Readers were blinded to each other and the AI-generated results. The 20 randomly selected patients were the same for all readers. To ensure feasibility within a reasonable amount of time, only the three most successful SPECT/CT markers in the previous analysis were included. Based on the change in uptake pre-to-post-treatment, the SUV_max_ of the LV and based on the association with functional parameters, the SUV_peak_ of the myocardium was included in the reader study. For the amyloid-affected volume of the RV (based on the association with outcomes), requiring comparatively lengthy manual delineation of the RV, AI-based assessment was compared with manual delineation times from literature. The inter-rater variability of the clinical readers was assessed using the intraclass correlation coefficient (ICC).

### Statistical analysis

Continuous variables are displayed as median with interquartile range (IQR) or as mean ± standard deviation (SD). Categorical data are shown as counts with associated percentages. Assessment of normality was performed using the Shapiro-Wilk test. The comparison of independent groups was performed using the Mann-Whitney U test for non-normal parameters and a t-test for independent samples if normality was given based on the Shapiro-Wilks test. For the clinical characteristics assessment, categorical parameters were tested using the Chi-square or Fisher’s exact test. Pairwise comparisons for patients before and after disease-modifying therapy were performed using the Wilcoxon signed-rank test for the non-parametric scenario and paired t-tests for normally distributed parameters. Time-to-event analysis was performed using Kaplan-Meier estimators, log-rank tests, and Cox proportional hazards models adjusted for age, sex and treatment type as well as changes in troponin T, NT-proBNP, 6-minute walk time distance and NYHA class. For the time-to-event analysis, patients were stratified based on whether parameters were increasing or decreasing post- versus pre-treatment. The clinical endpoint was death or heart failure-associated hospitalization. Two-sided *p-*values less than 0.05 were considered significant. All *p* values are to be interpreted exploratorily. The statistical analysis was performed using Python 3.9.5.

## Results

### Patient characteristics

**Fig. 1:**
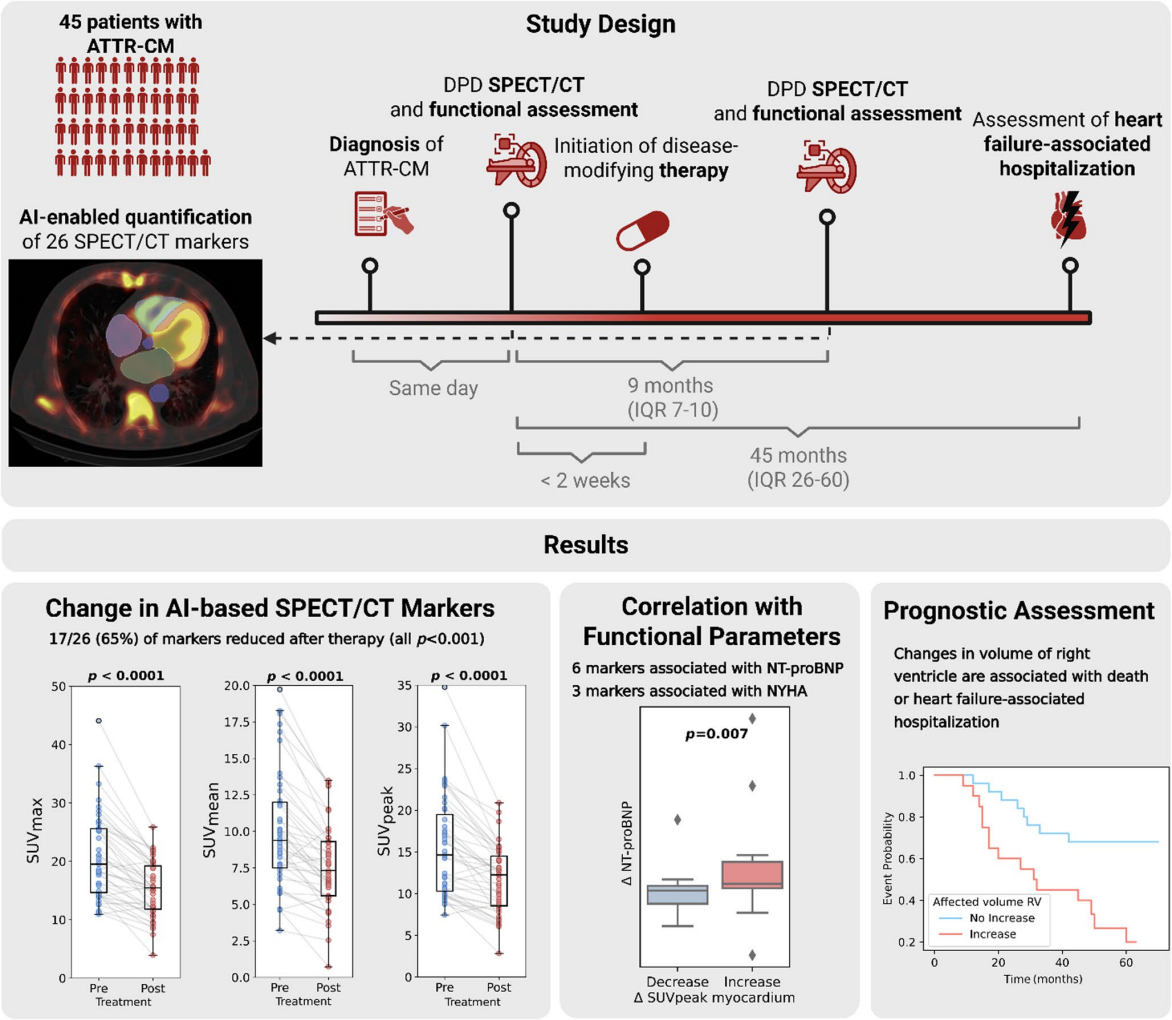
Graphical abstract detailing the design and main results of the study. Reduction in artificial intelligence (AI) assessed SPECT/CT markers after versus before treatment was associated with functional parameters and clinical outcomes

**Fig. 2:**
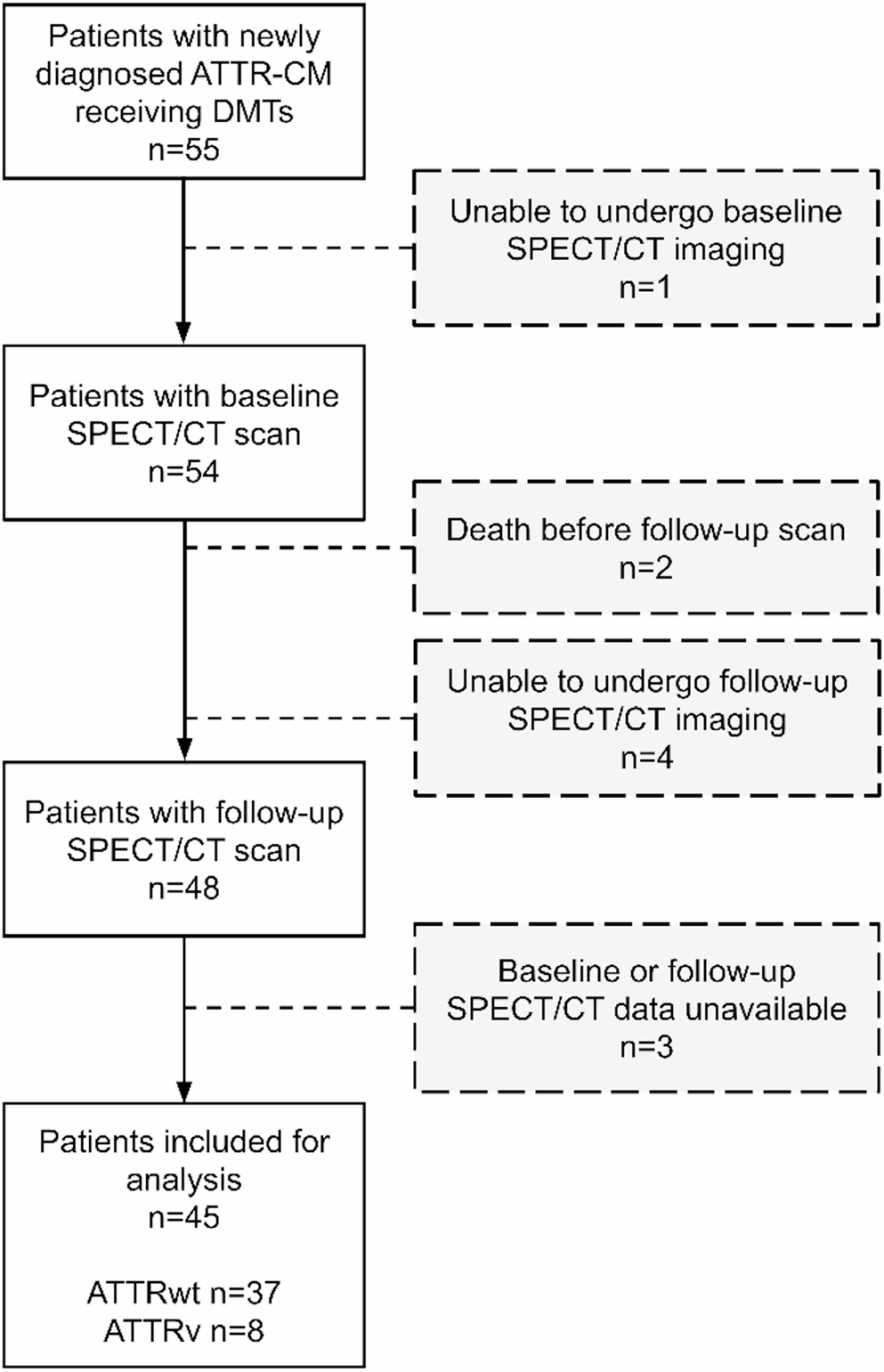
Cohort flow diagram

**Fig. 3:**
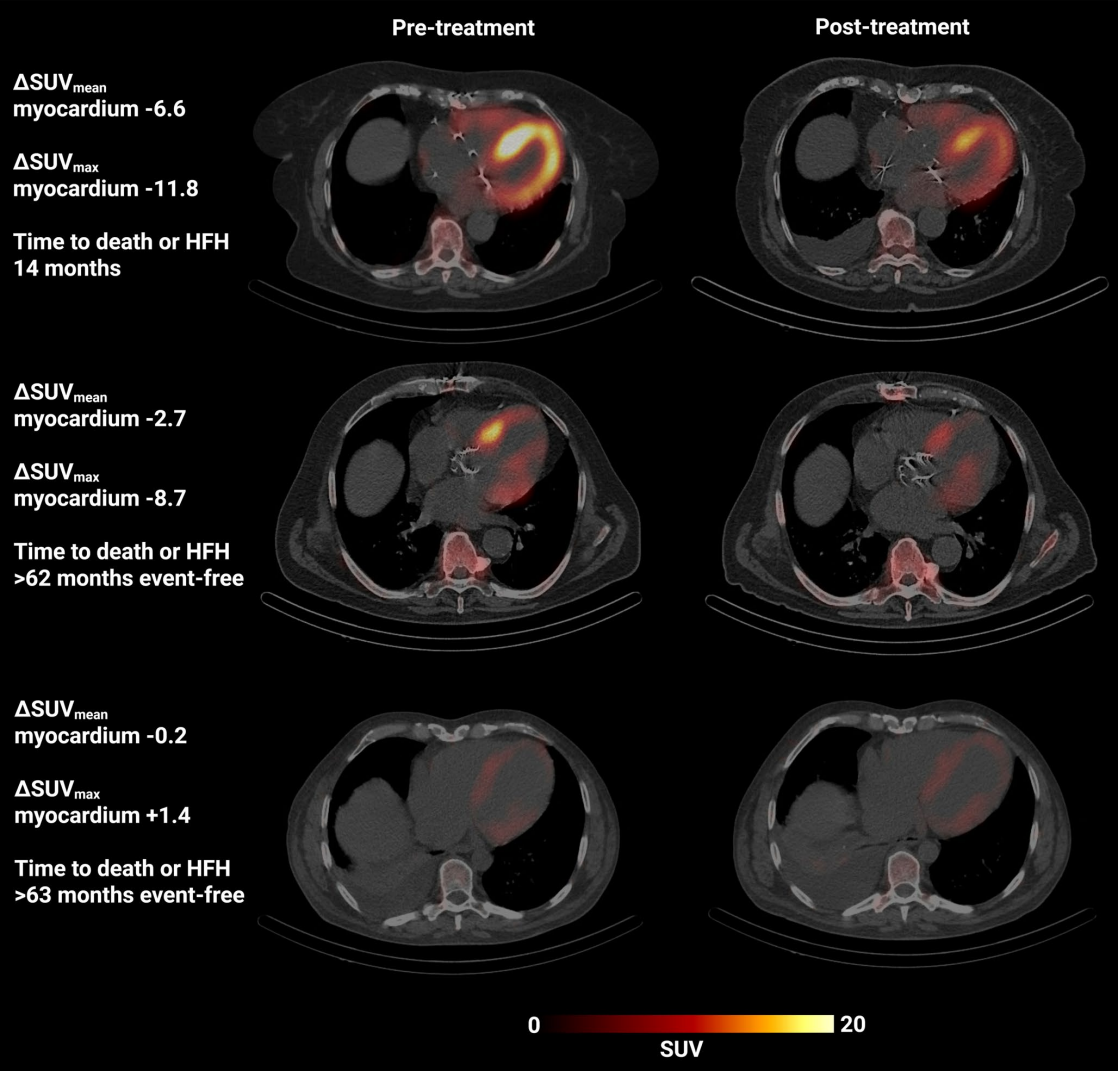
Representative SPECT/CT images of three patients (rows) before and after treatment

This study analyzed longitudinal data from 45 ATTR-CM patients with a median time between baseline and follow-up scans of 9 months (IQR 7–10). The study included 37 ATTRwt-CM patients receiving the transthyretin stabilizer tafamidis and 8 ATTRv-CM patients of whom 5 received RNA interference therapy via patisiran and 3 of whom received the antisense oligonucleotide inotersen. The mean age of the cohort was 77 ± 10 years, with the majority being male (82%, *n* = 37). For the assessment of patient characteristics, patients were stratified based on LV SUV_max_ response to DMTs into two groups: those with reduced LV SUV_max_ (Δ LV SUV_max_ < 0; 89%; *n* = 40) and those without a reduction (Δ LV SUV_max_ ≥ 0; 11%; *n* = 5). The mean age was slightly higher in the reduced uptake group (77 ± 10 years) compared to the non-reduced uptake group (72 ± 10 years), though this difference was not statistically significant (*p* = 0.33). Two patients died before the follow-up SPECT/CT scan. Clinical characteristics are displayed in Table 1. An overview of the study is shown in Fig. 1 and a cohort flow diagram is shown in Fig. 2. Representative images are shown in Fig. 3.

**Table 1:** Clinical characteristics

Parameter	Value	Entire data	Not reduced uptake (SUV_max_ LV)	Reduced uptake (SUV_max_ LV)	*P* value
Number of patients	n (%)	45 (100.0%)	5 (11.1%)	40 (88.9%)	
Age	Years	76.8 (9.8)	71.7 (9.5)	77.3 (9.7)	0.33
Sex	Female	8 (17.8%)	1 (16.7%)	7 (17.5%)	1.00
	Male	37 (82.2%)	4 (80.0%)	33 (82.5%)	
	NA	3 (6.7%)	0 (0%)	3 (7.7%)	
Treatment	Tafamidis	37 (82.2%)	3 (60.0%)	34 (85.0%)	0.33
	Patisiran	5 (11.1%)	1 (20%)	4 (10%)	
	Inotersen	3 (6.7%)	1 (20%)	2 (5%)	
NYHA	1	5 (11.11%)	0 (0%)	5 (12.5%)	0.004
	2	11 (24.44%)	4 (80.0%)	7 (17.5%)	
	3	22 (48.89%)	0 (0%)	22 (55.0%)	
	4	0 (0%)	0 (0%)	0 (0%)	
	NA	7 (15.56%)	1 (20.0%)	6 (15.0%)	
6MWD		388.66 (131.92)	476.75 (75.69)	376.07 (133.44)	0.13
	NA	13 (28.89%)	1 (20.00%)	12 (30.00%)	
proBNP	pg/mL	3419 (4614)	3387 (2638)	3423 (4809)	0.61
	NA	1 (2.22%)	0 (0.00%)	1 (2.50%)	
Troponin T	ng/L	60.39 (51.02)	48.2 (14.19)	61.95 (53.75)	0.78
	NA	1 (2.22%)	0 (0.00%)	1 (2.50%)	
Δ NYHA	−1	10 (22.2%)	2 (40.0%)	8 (20.0%)	0.70
	−2	1 (2.2%)	0 (0%)	1 (2.5%)	
	0	26 (57.8%)	2 (40.0%)	24 (60.0%)	
	+ 1	1 (2.2%)	0 (0%)	1 (2.5%)	
	NA	7 (15.6%)	1 (20.0%)	6 (15.0%)	
Δ 6MWD		−4.53 (75.16)	−22.0 (38.88)	−2.04 (78.68)	0.63
	NA	13 (28.9%)	1 (20.0%)	12 (30.0%)	
Δ proBNP	pg/mL	167 (1677)	527.2 (1337.98)	120.47 (1710.36)	0.05
	NA	1 (2.2%)	0 (0.0%)	1 (2.5%)	
Δ Troponin T	ng/L	3.86 (11.92)	−1.0 (5.76)	4.51 (12.37)	0.24
	NA	3 (6.7%)	0 (0.0%)	3 (7.5%)	

*NYHA* New York Heart Association, *6MWD* 6-minute walk distance, *LV* left ventricle. Patients were stratified based on whether they had a reduced LV SUVmax (Δ LV SUVmax < 0; Reduced) or not (Δ LV SUVmax ≥ 0; not reduced)

### Manually derived markers and inter-rater variability

The manually assessed markers were strongly correlated with the AI-assessed markers for LV SUV_max_ (*R* = 0.83, *p* < 0.0001), myocardial SUV_peak_ (*R* = 0.78, *p* < 0.0001). While the AI-based assessments completely removed inter-rater variability, the manual assessment by the three readers was good but not perfect, with ICCs of 0.95 (*p* < 0.0001) for both, LV SUV_max_ and myocardial SUV_peak_, respectively. The manual assessment of the LV SUV_max_ and myocardial SUV_peak_ took an average of 31 ± 5 s. For the amyloid-affected volume of the RV, where time-consuming delineation of the RV is required, literature indicated that average manual slice-by-slice delineation times for the RV alone are 658 (~ 11 min) ± 111 s (~ 2 min) [20]. The AI-based extraction was substantially faster with 104 ± 31 s for the extraction of all 26 markers while 4.1 ± 0.1 s were attributable to the extraction of LV SUV_max_, 5.1 ± 0.1 s to SUV_peak_, and 4.6 ± 0.1 s to amyloid-affected volume of the RV.

### SPECT/CT markers and changes after treatment

**Fig. 4:**
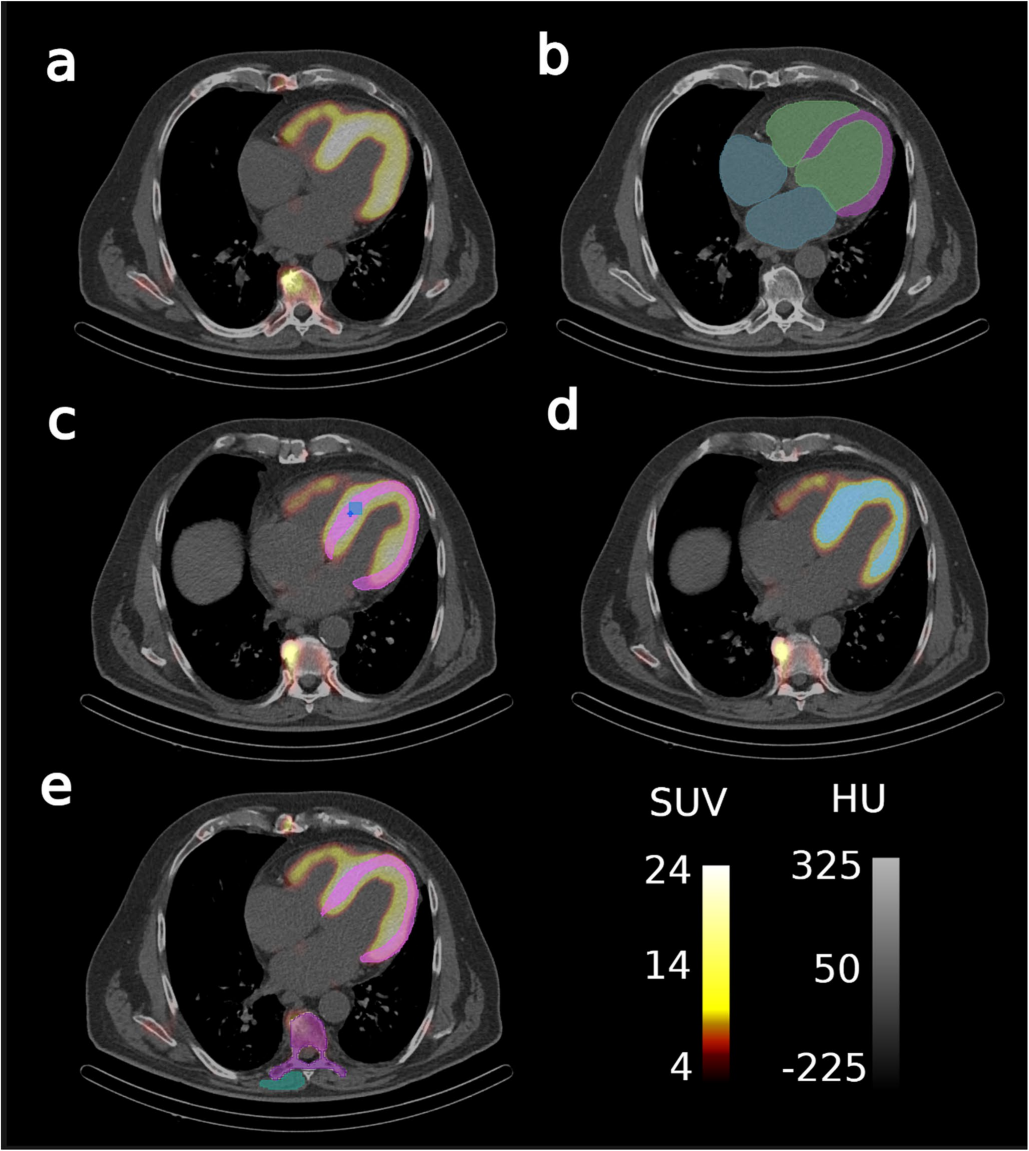
Example patient with segmentation masks for myocardium-based markers. (**a**) SPECT/CT, (**b**) CT with base segmentations, (**c**) segmentation for extraction of classical SUV parameters with SUV_peak_ shown in light blue and SUV_max_ shown in dark blue, (**d**) segmentation for amyloid-affected volume and amyloid activity, and (**e**) segmentations for retention index

The highest uptake at baseline was associated with the SUV_max_ of the myocardium (19.5 [IQR 14.9; 25.6]), followed by the SUV_max_ of the LV (18.6 [IQR 14.9; 23.2]) and the SUV_peak_ of the myocardium (14.6 [IQR 10.6; 18.9]). Example images with corresponding AI-segmented target regions and markers are shown in Fig. 4.

**Fig. 5:**
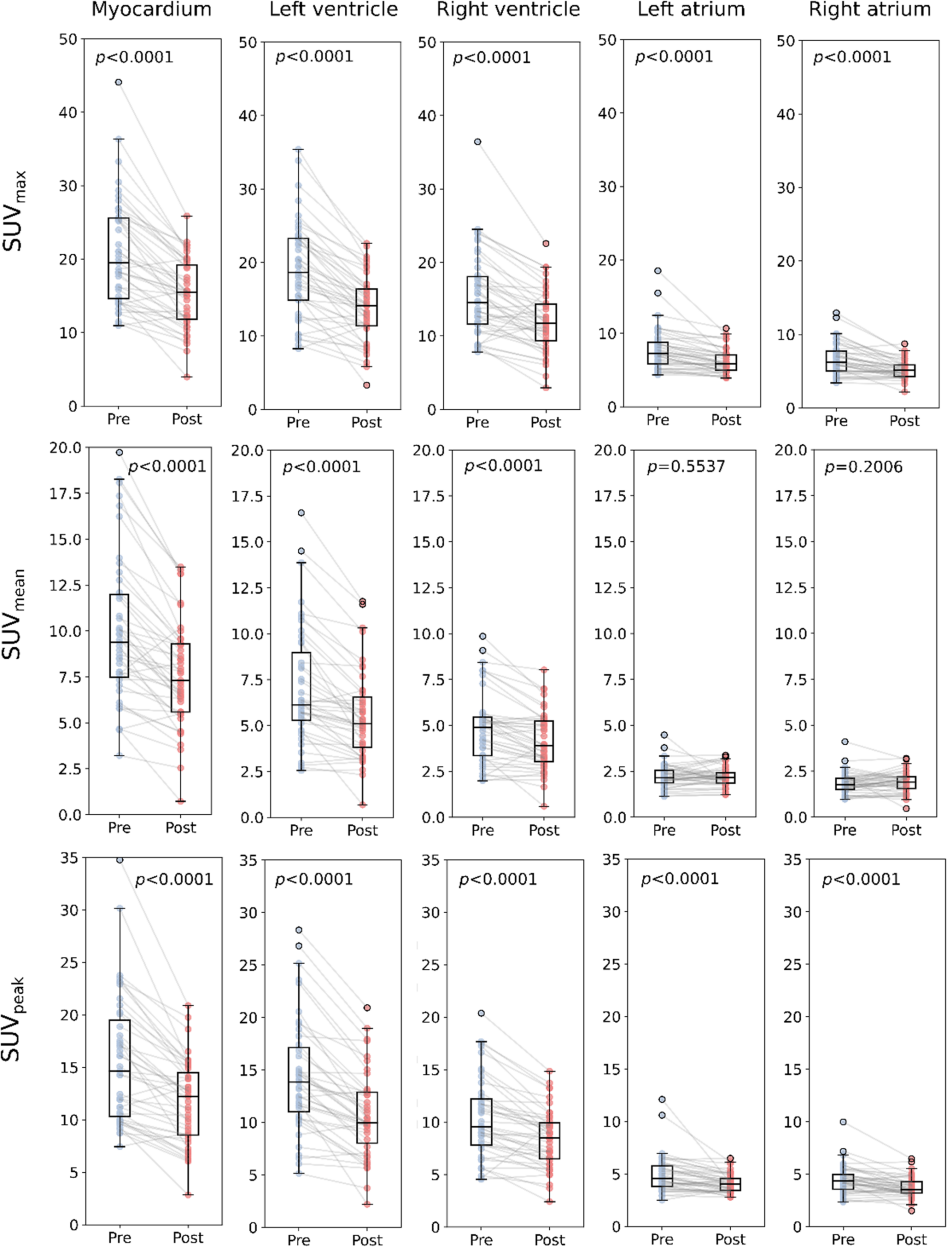
Changes in selected SPECT/CT markers pre- versus post-treatment for classical SUV markers extracted using AI. The terms ‘pre’ and ‘post’ refer to pre- and post-treatment markers

Post-treatment assessments revealed significant reductions in 14/16 (88%) SUV metric (max, mean, peak) markers and a significant reduction in the retention index (all *p* < 0.0001) while amyloid activity significantly decreased in 3/5 (60%) of markers (all *p* < 0.0001) and affected volume markers increased in 3/5 (60%) markers after treatment initiation, however not significantly (all *p* > 0.05). The non-significant SUV metric markers were limited to the SUV_mean_ of the left and right atria (*p* = 0.201 and *p* = 0.554, respectively). The marker with the highest absolute difference was the SUV_max_ of the LV with 4.5, resulting from a pre-treatment SUV_max_ of 18.6 (IQR 14.9; 23.3) and a post-treatment SUV_max_ of 14.1 (IQR 11.4; 16.4). SUV_max_ of the LV was closely followed by the SUV_max_ of the largely overlapping myocardium with an absolute difference of 4.0 (pre-treatment SUV_max_ 19.5 [IQR 14.7; 25.6]; post-treatment SUV_max_ 15.5 [IQR 11.8; 19.2]). Visual representations of the changes in SUV metric-based markers are shown in Fig. 5 and the corresponding quantitative results are shown in Table 2.

**Table 2:** Changes in SPECT/CT markers pre- versus post-treatment

Parameter	*P* value	Pre-treatment median (IQR)	Post-treatment median (IQR)
SUVmax LV	< 0.0001	18.6 (14.9; 23.3)	14.1 (11.4; 16.4)
SUVmax myocardium	< 0.0001	19.5 (14.7; 25.6)	15.5 (11.8; 19.2)
SUVpeak LV	< 0.0001	13.9 (11.0; 17.1)	10.0 (8.0; 12.9)
SUVpeak myocardium	< 0.0001	14.6 (10.3; 19.5)	12.2 (8.5; 14.5)
Amyloid activity myocardium	< 0.0001	302.4 (229.2; 420.0)	239.5 (187.2; 322.0)
Amyloid activity LV	< 0.0001	124.4 (101.5; 191.0)	97.5 (75.2; 139.2)
SUVmax LA	< 0.0001	7.3 (5.8; 8.8)	5.8 (5.0; 7.1)
SUVmean LV	< 0.0001	6.1 (5.3; 9.0)	5.1 (3.8; 6.6)
SUVmean myocardium	< 0.0001	9.4 (7.5; 12.0)	7.3 (5.6; 9.3)
SUVmax RV	< 0.0001	14.6 (11.6; 18.1)	11.7 (9.3; 14.3)
SUVmax RA	< 0.0001	6.2 (5.0; 7.7)	5.1 (4.3; 5.8)
SUVpeak RV	< 0.0001	9.5 (7.8; 12.2)	8.5 (6.5; 9.9)
SUVmean RV	< 0.0001	4.9 (3.4; 5.4)	3.9 (3.0; 5.3)
Retention index	< 0.0001	4.4 (3.2; 7.2)	3.1 (2.4; 4.8)
Amyloid activity RV	< 0.0001	102.9 (75.7; 149.1)	89.4 (65.1; 123.7)
SUVpeak LA	< 0.0001	4.6 (3.8; 5.8)	4.0 (3.4; 4.6)
SUVpeak RA	< 0.0001	4.4 (3.6; 5.0)	3.5 (3.2; 4.3)
SUVmean RA	0.2006	1.8 (1.5; 2.1)	1.9 (1.6; 2.2)
Affected volume myocardium	0.3817	92.6 (60.3; 120.4)	93.8 (70.9; 115.3)
Amyloid activity LA	0.3917	29.7 (22.6; 35.9)	26.3 (22.1; 33.4)
SUVmean LA	0.5537	2.1 (1.9; 2.6)	2.2 (1.9; 2.4)
Affected volume RA	0.6712	96.6 (69.7; 122.5)	90.2 (71.4; 119.6)
Amyloid activity RA	0.6862	24.4 (18.4; 33.7)	26.5 (20.3; 32.7)
Affected volume LA	0.7176	74.8 (65.2; 85.7)	77.5 (64.1; 85.8)
Affected volume RV	0.7333	118.6 (96.5; 135.7)	120.3 (102.5; 139.9)
Affected volume LV	0.8477	97.6 (85.8; 110.1)	92.8 (81.8; 115.1)

Parameters are sorted by descending *p* value

### Differences in treatment groups

Compared to other treatments, patients who received tafamidis exhibited a significant reduction in NT-proBNP levels (−105 vs. +1391, *p* = 0.03), a markedly greater decrease in myocardial SUV_max_ (−6.55 vs. −2.14, *p* = 0.003), LV SUV_max_ (−6.25 vs. −1.40, *p* < 0.001), myocardial SUV_mean_ (−2.72 vs. −1.03, *p* = 0.02), myocardial SUV_peak_ (−4.45 vs. −1.67, *p* = 0.02), LV SUV_peak_ (−4.26 vs. −1.22, *p* = 0.006), and amyloid activity of the myocardium (−88.86 vs. −29.43, *p* = 0.007). The remaining clinical baseline characteristics and changes in SPECT/CT markers did not differ between the treatment groups.

### Association of changes in SPECT/CT markers with functional parameters

**Fig. 6:**
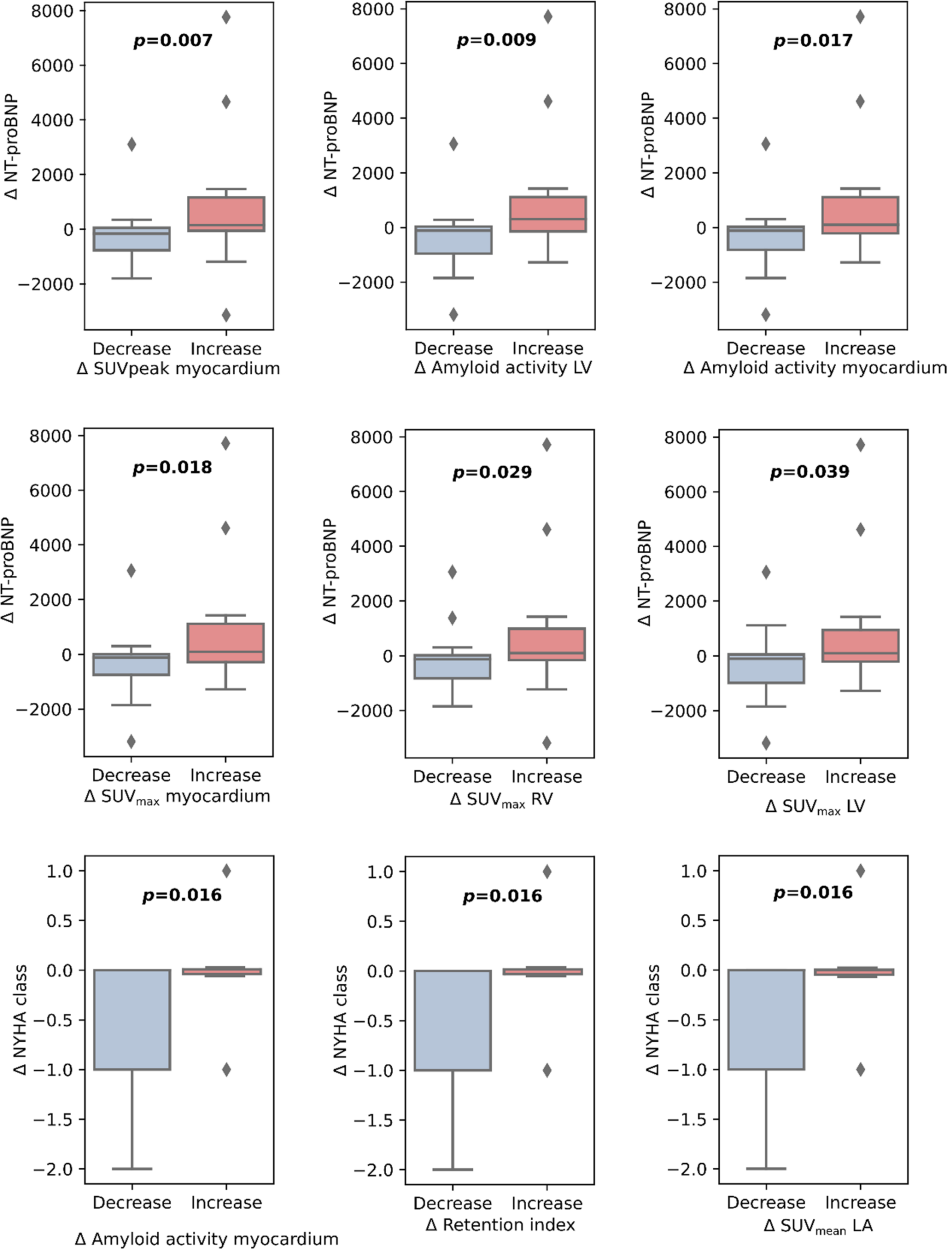
Association of changes in SPECT/CT markers with functional parameters before versus after treatment for NT-proBNP (upper two rows) and New York Heart Association grading (NYHA, lower row)

The change in SPECT/CT markers after versus before treatment corresponded to improvements in functional parameters. This included improved NT-proBNP levels for patients with reduced SUV_peak_ of the myocardium (*p* = 0.007), amyloid activity of the LV (*p* = 0.009), amyloid activity of the myocardium (*p* = 0.02), SUV_max_ of the myocardium (*p* = 0.02), SUV_max_ of the RV (*p* = 0.03), and SUV_max_ of the LV (*p* = 0.04). Reduced amyloid activity of the myocardium, retention index, and SUV_mean_ of the LA were associated with improvements in New York Heart Association (NYHA) stage (all *p* = 0.016). Visual representations of the associations between changes in SPECT/CT markers and functional parameters are shown in Fig. 6.

### Association of SPECT/CT markers with outcome

**Fig. 7:**
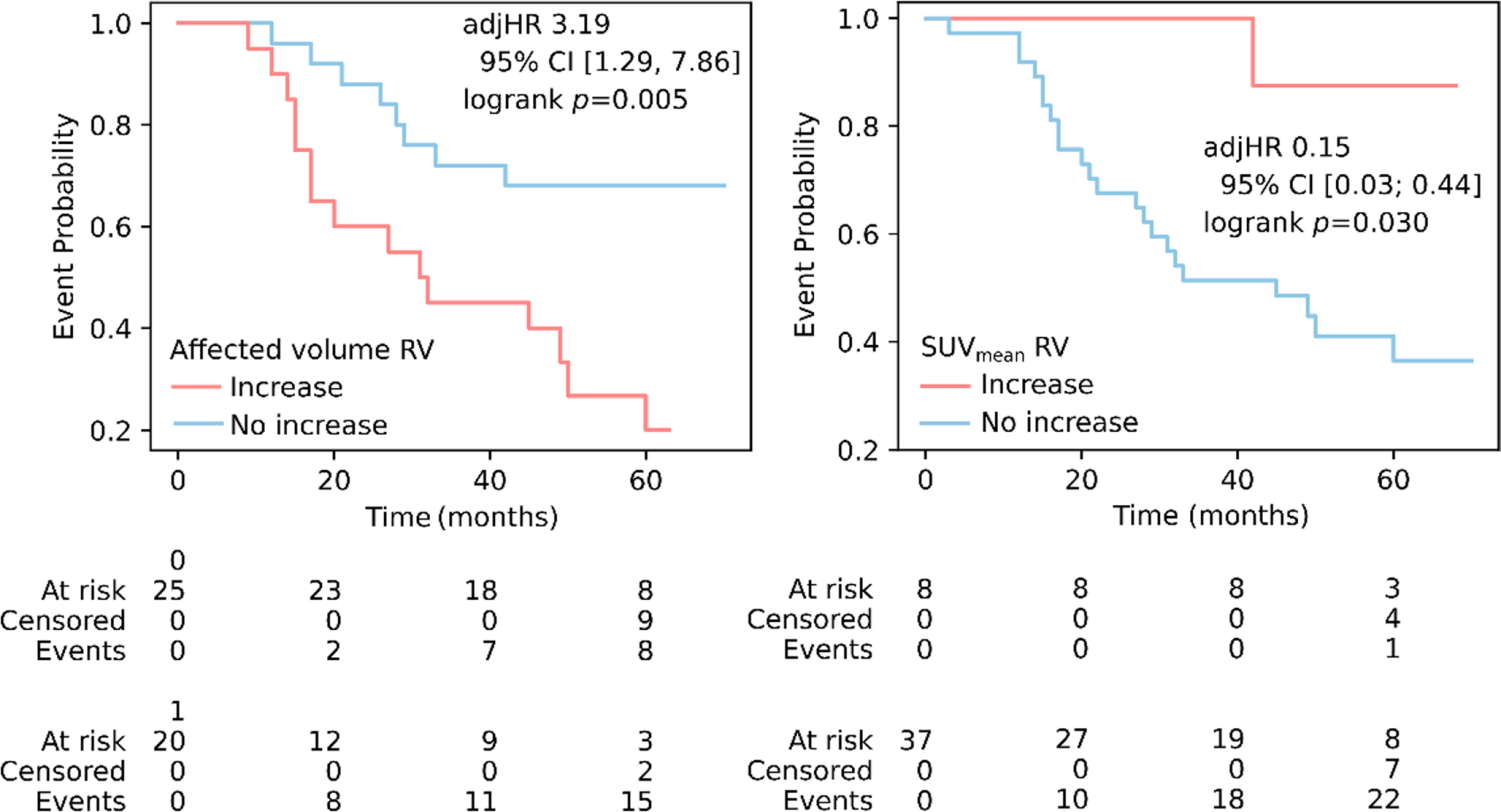
Association of death or heart failure-associated hospitalization with changes in amyloid-affected volume in the right ventricle (RV)

Follow-up information was available for all patients. After a median of 45 (IQR 26; 60) months, 23/45 (51%) patients were either hospitalized due to heart failure or had died. An increase of amyloid-affected volume in the RV (adjHR 3.19, 95% CI [1.29; 7.86], logrank *p* = 0.005) was associated with death or heart failure-associated hospitalization univariately and remained significant after adjustment for age, sex and treatment type as well as changes in troponin T, NT-proBNP, 6-minute walk time distance and NYHA class (Fig. 7). Further, the decrease in SUV_mean_ RV was associated with worse prognosis (adjHR 0.15 95% CI [0.02;1.10], logrank *p* = 0.030) before and after multivariate adjustment. Results on the association with outcome are shown in Table 3.

**Table 3:** Association of SPECT/CT markers with a composite outcome of heart failure-associated hospitalization or death. Adjusted hazard ratios (adjHR) show hazard ratios after correction for age, sex and treatment type as well as changes in troponin T, NT-proBNP, 6-minute walk time distance and NYHA class

Marker	Univariate HR (95% CI)	Univariate *p* value (log-rank)	Multivariate HR (95% CI)	Multivariate *p* value (Cox)
Δ Affected volume RV	3.19 (1.29; 7.86)	0.005	3.12 (1.34; 7.30)	0.008
Δ SUVmean RV	0.15 (0.02; 1.10)	0.030	0.17 (0.03; 0.44)	0.006
Δ Affected volume RA	2.14 (0.90; 5.07)	0.077		
Δ Retention index	0.38 (0.11; 1.26)	0.100		
Δ SUVmax LA	0.24 (0.03; 1.79)	0.131		
Δ SUVmean LA	0.56 (0.25; 1.28)	0.164		
Δ SUVmean LV	0.30 (0.04; 2.26)	0.215		
Δ SUVmean RA	0.61 (0.27; 1.38)	0.229		
Δ SUVpeak RV	0.31 (0.04; 2.33)	0.230		
Δ SUVmax myocardium	0.34 (0.05; 2.51)	0.265		
Δ Amyloid activity RA	0.63 (0.27; 1.46)	0.277		
Δ SUVmax RV	0.35 (0.05; 2.58)	0.277		
Δ SUVmax RA	0.52 (0.15; 1.75)	0.282		
Δ SUVpeak RA	0.63 (0.25; 1.61)	0.334		
Δ Amyloid activity RV	0.57 (0.17; 1.93)	0.363		
Δ Amyloid activity LV	0.52 (0.12; 2.21)	0.364		
Δ SUVmax LV	0.47 (0.06; 3.48)	0.448		
Δ SUVpeak LV	0.47 (0.06; 3.48)	0.448		
Δ Affected volume LV	1.35 (0.59; 3.09)	0.474		
Δ SUVpeak myocardium	0.51 (0.07; 3.83)	0.510		
Δ Affected volume LA	1.30 (0.57; 2.96)	0.529		
Δ Amyloid activity LA	0.82 (0.36; 1.87)	0.633		
Δ SUVmean myocardium	1.28 (0.30; 5.54)	0.733		
Δ Amyloid activity myocardium	0.83 (0.19; 3.53)	0.799		
Δ SUVpeak LA	1.10 (0.43; 2.79)	0.847		
Δ Affected volume myocardium	1.01 (0.44; 2.29)	0.985		
Δ NYHA class	5.58 (1.39; 22.47)	0.007	8.98 (1.78; 45.18)	0.008
Δ Troponin T	0.73 (0.32; 1.65)	0.450		
Treated with tafamidis	0.70 (0.26; 1.89)	0.500		
Δ 6MWD	0.76 (0.33; 1.75)	0.514		
Δ NT-proBNP	1.04 (0.46; 2.34)	0.921		

## Discussion

This study demonstrates that AI-enabled [^99m^Tc]Tc-DPD SPECT/CT imaging markers enable robust, reproducible, and efficient quantification of treatment response in patients with ATTR-CM undergoing DMT. We further show that the extracted imaging markers are associated with functional parameters and outcome. Moreover, in a comparison with imaging physicians, our results demonstrate a strong consistency of the most important AI-extracted markers with physician assessment while AI-based marker extraction reduces assessment times and removes inter-rater variability.

Among the 26 imaging biomarkers derived through automated segmentation, 65% showed a significant decline following treatment, indicating that SPECT/CT markers reflect a measurable response to therapy. These changes were particularly pronounced in SUV metrics of the LV and myocardium but were also present in other cardiac subregions. In contrast, affected-volume parameters did not show significant changes following treatment. This finding aligns with the evidence suggesting that currently approved disease-modifying therapies do not actively remove existing amyloid deposits but instead primarily reduce the deposition of new amyloid as well as tracer activity [12, 13, 21, 22]. As such, SUV-based and amyloid (DPD) activity markers, reflecting ongoing tracer uptake, are more sensitive to short-term therapeutic effects than volume-based measures.

Several of the AI-extracted markers were associated with established functional and clinical indicators of disease severity and prognosis. Decreases in SUV_peak_ and amyloid activity were significantly associated with improvements in NT-proBNP and NYHA class. Moreover, increased amyloid-affected volume in the RV was independently associated with adverse outcomes, including death or heart failure hospitalization, underscoring the prognostic value of regional SPECT/CT markers. While changes in RV affected volume and SUV_mean_ RV were associated with prognosis, LV markers parameters, including those based on SUV, were not. This may reflect that RV involvement indicates more advanced biventricular disease and right-sided failure, both strong prognostic factors in cardiac amyloidosis [23, 24]. Further, LV SUV metrics may be more affected by partial-volume effects, higher baseline uptake, and motion artifacts, which can reduce their dynamic range and prognostic sensitivity. RV affected volume captures spatial extension of tracer uptake and may better reflect progressive amyloid infiltration. Interestingly, a decrease in SUV_mean_ RV was associated with worse prognosis. While the number of patients with an increase of SUV-based markers during therapy was small and results were not significant for other SUV markers, a decrease was associated with worse prognosis (HR < 1.00) for most SUV markers.

Our findings support the potential of AI-enabled SPECT/CT as a non-invasive imaging tool for the early assessment of treatment efficacy in ATTR-CM. While previous studies have described the potential utility of amyloid-avid tracers like [^99m^Tc]Tc-DPD for assessing response to treatment in ATTR-CM, primarily in planar scintigraphy but also via SPECT [12, 13, 22, 25, 26], our results provide evidence that imaging assessment can be performed in a more comprehensive fashion using multiple standardized and quantitative metrics that are extracted in a fraction of the time required for the assessment by human expert readers. Using the AI-based metrics further increased reproducibility by eliminating inter-rater variability, often a limitation of manual assessment [14, 27–29].

From a treatment-specific perspective, patients treated with tafamidis exhibited larger reductions in both tracer uptake and NT-proBNP compared to those receiving RNA-targeting therapies. While this may reflect differences in disease stage or patient selection, as tafamidis-treated patients were significantly older, the consistency of response across multiple SPECT/CT parameters suggests that the observed changes are generalizable across treatments.

Future studies should explore how changes in these imaging biomarkers relate to long-term clinical endpoints in randomized controlled trials. Moreover, integrating AI-derived imaging biomarkers with multi-omics and longitudinal clinical data could open new pathways for truly personalized disease management in ATTR-CM. While this study is concerned with assessing the developed markers for treatment response assessment, the markers may also be evaluated for diagnosis, prognosis, subtyping, and treatment decision-making and may even be applied to PET imaging with other tracers [11, 30, 31]. Further, the markers developed in this study may be extended, for example, by integrating AI-enabled markers that quantify cardiac anatomy or markers related to tracer activity beyond the heart, both of which have recently been shown to provide valuable information in cardiovascular disease [15, 32, 33]. Lastly, novel treatments currently in clinical trials have different mechanisms of action compared to currently approved DMTs, potentially depleting existing amyloid deposits [34]. The effect of such treatments may be investigated using the showcased quantitative metrics and compared to their changes introduced by currently employed DMTs identified in this study.

Several limitations inherent to this study should be acknowledged. While, to the best of our knowledge, this is the most comprehensive study for the assessment of SPECT/CT for treatment response evaluation in ATTR-CM based on the number of included patients, the sample size is still moderate. Although the findings were statistically robust, larger multicentric cohorts are needed to confirm the generalizability of the developed imaging biomarkers, particularly for treatments other than tafamidis. While we did not identify any differences in baseline parameters between patients who received tafamidis and those who received other treatments, the collected parameters may not fully represent baseline disease stage. Considering this, as well as the small sample size of the non-tafamidis group, the greater reduction of SPECT/CT markers in the tafamidis treatment group may reflect baseline disease stage rather than treatment effect. Additionally, while AI-based segmentation was rigorously validated, the potential for systematic bias in image registration between the CT component and the associated SPECT modality as well as the associated tracer quantification across different scanners and protocols requires further exploration. While this study focused on [^99m^Tc]Tc-DPD, the AI-based segmentation and quantification pipeline operates independently of the specific bone-avid tracer, as it relies on standardized uptake measures and anatomical CT guidance. Prior comparative work has shown similar myocardial distribution and diagnostic performance among [^99m^Tc]Tc-DPD, technetium-99m pyrophosphate ([^99m^Tc]Tc-PYP), and technetium-99m hydroxymethylene diphosphonate ([^99m^Tc]Tc-HMDP). Nevertheless, tracer-specific differences, including soft-tissue kinetics and binding affinity could necessitate calibration when applying the derived metrics to other tracers. Future multicentric efforts including tracers beyond [99mTc]Tc-DPD will therefore be important to validate cross-tracer generalizability and ensure quantitative harmonization. In addition, future studies may further evaluate the association of the AI-derived SPECT/CT markers with cardiac magnetic resonance imaging (MRI) parameters and echocardiographic parameters.

## Conclusion

This study underscores the utility of AI-enhanced [^99m^Tc]Tc-DPD SPECT/CT imaging in capturing treatment-associated changes in tracer activity and uptake patterns. By automating marker extraction, AI mitigates inter-rater variability, increases efficiency, and enhances inter-rater reproducibility. Our findings highlight the potential of SPECT/CT markers to serve as sensitive, non-invasive biomarkers for treatment response assessment, offering an alternative to cardiac MRI in patients with implanted devices. These findings pave the way for personalized treatment strategies and broader clinical adoption of AI in nuclear medicine and molecular imaging.

## Data Availability

Data is available from the corresponding author upon reasonable request.
